# Supplementary material for: HIF-1α-mediated feedback prevents TOR signalling from depleting oxygen supply and triggering stress during normal development
Source: Nat Commun. 2025 Dec 21;17:397. doi: 10.1038/s41467-025-67089-6 (PMC12796383; doi:10.1038/s41467-025-67089-6)

SUPPLEMENTARY INFORMATION

HIF-1α-mediated feedback prevents TOR signalling from depleting oxygen supply and triggering stress during normal development

Yifan Zhao[1], Cyrille Alexandre[1], Gavin Kelly[1], Jean-Paul Vincent[1*], & Gantas Perez-Mockus[1*]

[1]The Francis Crick Institute, London, UK

[*]These authors jointly supervised this work

gantas.perezmockus@crick.ac.uk

jp.vincent@crick.ac.uk

Table of Contents

Supplementary Figure 1 1

Supplementary Figure 2 3

Supplementary Figure 3 5

Supplementary Figure 4 6

Supplementary Figure 5 8

Supplementary Figure 6 10

Supplementary Figure 7 12

Supplementary Figure 8 14

Supplementary Figure 9 16

References 18

Source Data for Supplementary Figure 6a 19

Source Data for Supplementary Figure 6a' 20

Source Data for Supplementary Figure 6c 21

Supplementary Figure 1

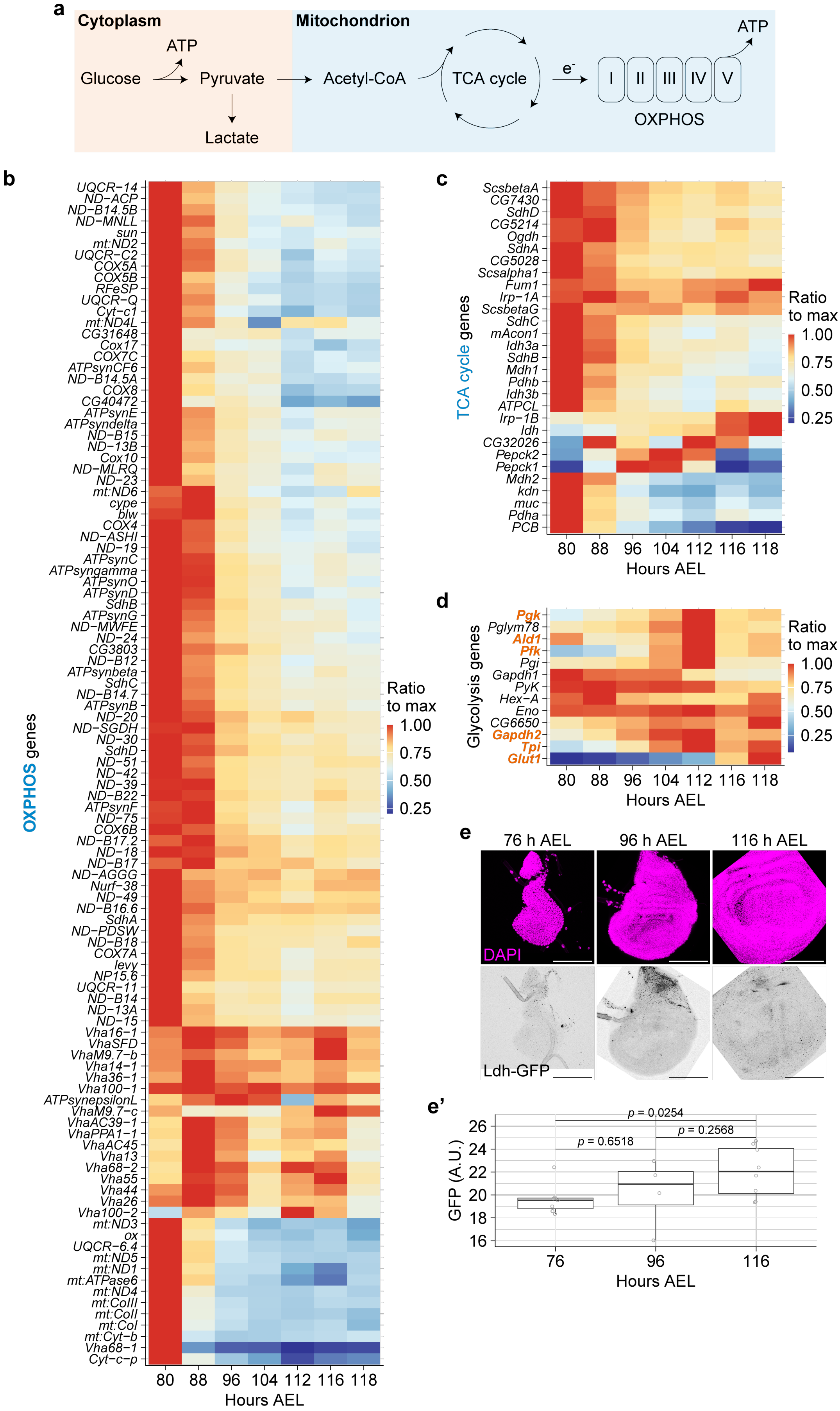

Supplementary Fig. 1: Transcriptomic analysis of L3 wing discs reveals transcriptional alterations typically associated with hypoxia.

a. Schematic representation of aerobic (blue shading) and anaerobic (orange shading) metabolism.
b. Detailed transcriptional profile of OXPHOS genes shown in **Fig. 1f** (with gene names). Source data are provided as an Excel sheet.
c. Transcriptional profile of TCA cycle genes during L3. Source data are provided as an Excel sheet.
d. Transcriptional profile of glycolysis genes during L3. The drop in expression at the end of L3 has been previously documented [1], probably due to cessation of feeding at the wandering stage. Expression of *Pgk*, *Ald1*, *Pfk*, *Tpi* and *Glut1* is known to be upregulated under hypoxia. Source data are provided as an Excel sheet.
e-e'. *Ldh* expression, assayed with Ldh-GFP increases in wing discs during L3. Representative images are shown in **e** and quantification of wing pouch fluorescence intensity is shown in **e'**. Scale bar = 100 μm. n $\geq$ 7 for each time point, except for data at 96 h AEL where n = 4. Normality of the data was confirmed using the Shapiro-Wilk test. Statistical significance was assessed by a two-sided unpaired t-test (p $\geq$ 0.05, not significant). In all box plots shown here and in subsequent figures, the centre line represents the median (50th percentile); the box limits indicate the lower (Q1, 25th percentile) and upper quartiles (Q3, 75th percentile); the interquartile range (IQR) is defined as $Q3 - Q1$; whiskers extend to data points within $1.5 \times IQR$ from the quartiles; data points outside this range are shown as outliers.

Supplementary Figure 2

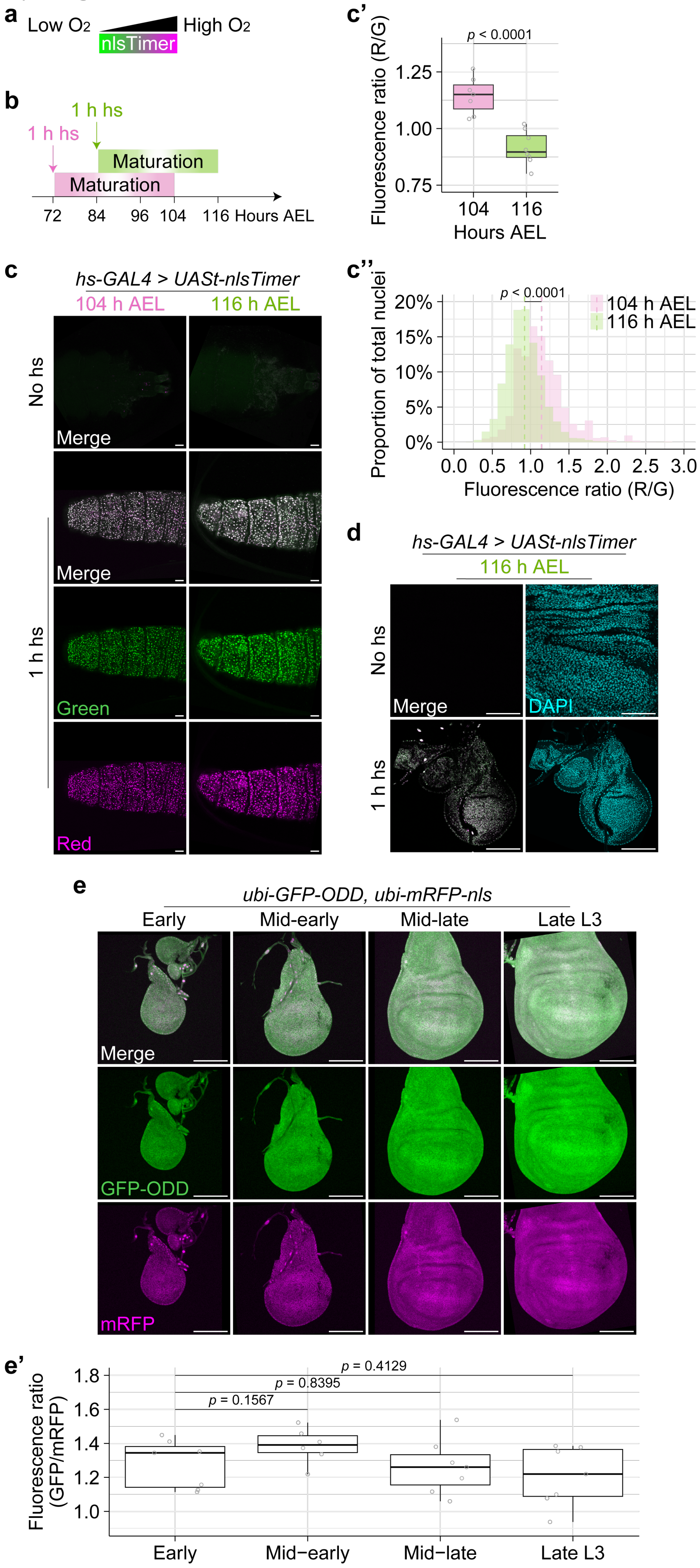

Supplementary Fig. 2: Behaviour of previously developed oxygen reporters during L3.

a. Schematic representation of nlsTimer's maturation in different oxygen levels. At low oxygen levels, the fluorophore matures to a green variant, whereas at high oxygen levels, it matures to a red variant.
b. Experimental protocol to compare oxygen levels in young and old L3 larvae with nlsTimer. Both 72 h and 84 h AEL larvae were simultaneously subjected to a heat shock treatment at 37 °C for 1 hour to induce a pulse of nlsTimer expression. The nlsTimer was then allowed to mature for 31 hours *in vivo* before imaging.
c-c". Red and green fluorescence from nlsTimer in L3 larvae at 104 and 116 h AEL. Red/green overlap is converted to white with FIJI in the merge. Representative images are shown in **c**. Scale bar = 100 μm. Quantification of nuclear fluorescence intensity (**c'** and **c"**) shows that the red fluorescence is relatively higher than the green fluorescence in younger larvae. n $\geq$ 7 for each time point. After confirming normality of the data, statistical significance was assessed by a two-sided unpaired t-test in **c'**. Statistical significance of the two histograms in **c"** was assessed by a two-sided unpaired Kolmogorov Smirnov test (K-S test) (Distance = 0.3314). Source data are provided as an Excel sheet.
d. Heat-shock-induced nlsTimer expression (by *hs-GAL4* > *UASt-nlsTimer*) impairs growth in wing discs. Merge indicates overlap of red and green signal. Scale bar = 100 μm.
e-e'. Fluorescence from GFP-ODD in L3 discs remains unchanged in environmental normoxia. Representative images are shown in **e** and quantification of wing pouch fluorescence intensity is shown in **e'**. Scale bar = 100 μm. n = 7 for each developmental stage, except for data at mid-early L3 where n = 6. After confirming normality of the data, statistical significance was assessed by a two-sided unpaired t-test.

Supplementary Figure 3

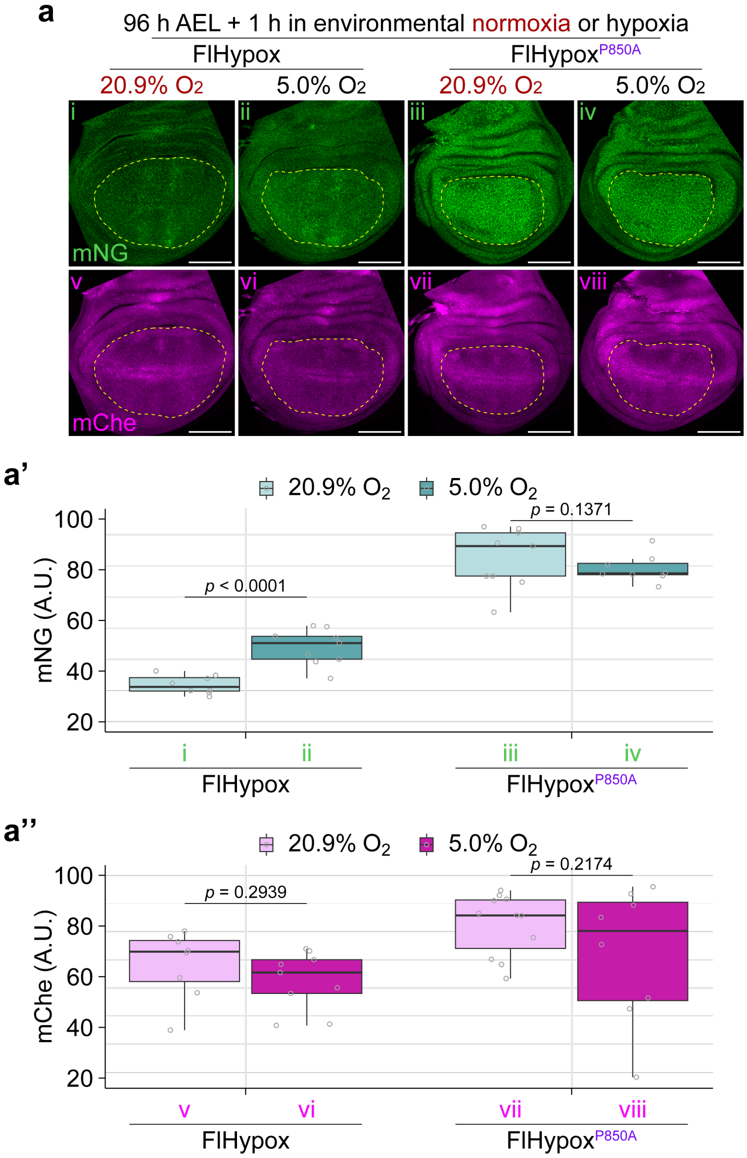

Supplementary Fig. 3: Comparing the activity of FlHypox and FlHypox^{P850A} in environmental hypoxia and normoxia.

a. Fluorescence from mNeonGreen and mCherry in the discs shown in **Fig. 2b**. Scale bar = 100 μm.

a'-a''. Quantification of mNeonGreen and mCherry fluorescence (shown as box plots aligned with the micrographs). n $\geq$ 8 for each condition. After confirming normality of the data, statistical significance was assessed by a two-sided unpaired t-test.

Supplementary Figure 4

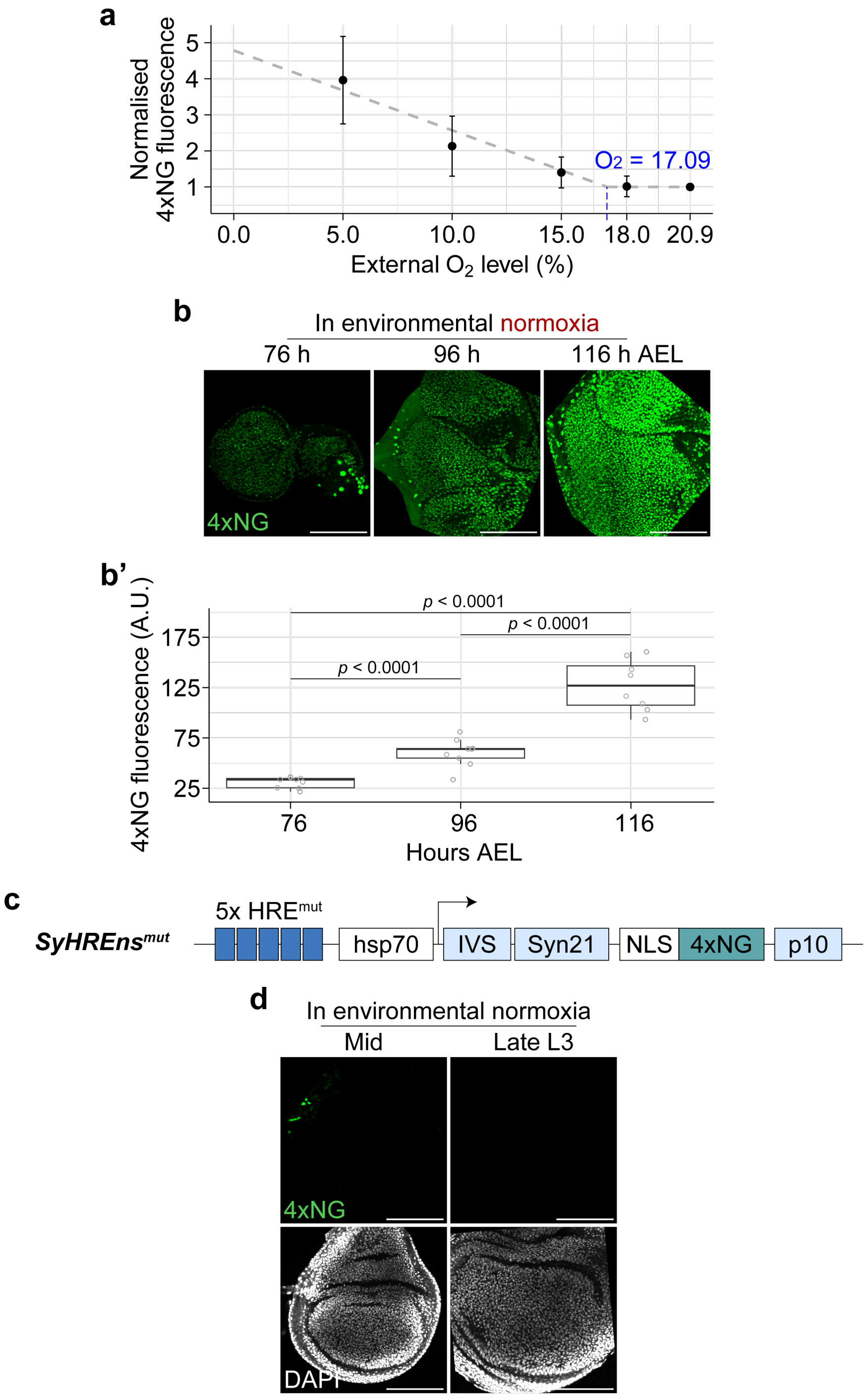

Supplementary Fig. 4: SyHREns and SyHREns^{mut} activity in various situations.

a. Quantification of SyHREns activity in discs from larvae cultured in different levels of oxygen for 2.5 h before imaging. Signal was normalised to the value in normoxia. There was no statistically significant difference between the values at 18 and 20.9%. Source data are provided as an Excel sheet.

b-b'. Fluorescence intensity from SyHREns in L3 eye discs increases under environmental normoxia. Representative images are shown in **b** and quantification is shown in **b'**. Scale bar = 100 μm. n = 9 for each time point, except for data at 116 h

AEL where n = 8. After confirming normality of the data, statistical significance was assessed by a two-sided unpaired t-test.

c. Schematics of $SyHREns^{mut}$ transgene and absence of signal in wing discs from transgenic larvae cultured in normoxia. Note that NLS4xNG is non-specifically expressed in tracheal cells. Scale bar = 100 μm.

Supplementary Figure 5

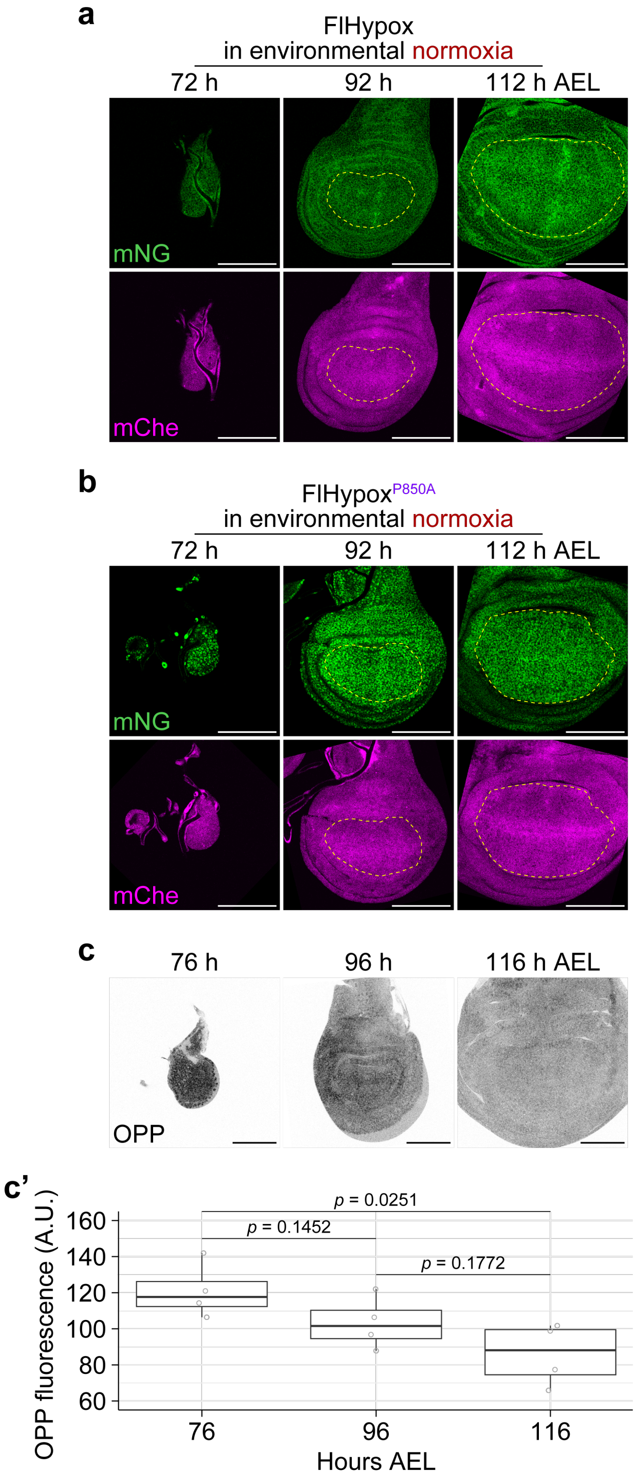

Supplementary Fig. 5: FlHypox and FlHypox$^{\mathbf{P850A}}$ activity and OPP incorporation in L3 wing discs.

a. FlHypox mNeonGreen fluorescence and mCherry fluorescence from the *ubi-mCherry* module at three different stages in L3 wing discs from larvae cultured in normoxia. The 92 h and 112 h AEL discs are the same samples as those shown in **Fig. 3c**. mNeonGreen fluorescence rises while mCherry fluorescence remains constant. Scale bar = 100 μm.

b. FlHypox^{P850A} mNeonGreen fluorescence and mCherry fluorescence from the *ubi-mCherry* module at three different stages in L3 wing discs from larvae cultured in normoxia. The 92 h and 112 h AEL discs are the same samples as those shown in **Fig. 3d**. Both signals remain unchanged over time. Scale bar = 100 μm.
c-c'. Representative images of O-propargyl-puromycin (OPP) incorporation by L3 discs at three different stages. Scale bar = 100 μm. Quantification in **c'** shows a progressive decrease with age. n = 4 for each time point. After confirming normality of the data, statistical significance was assessed by a two-sided unpaired t-test.

Supplementary Figure 6

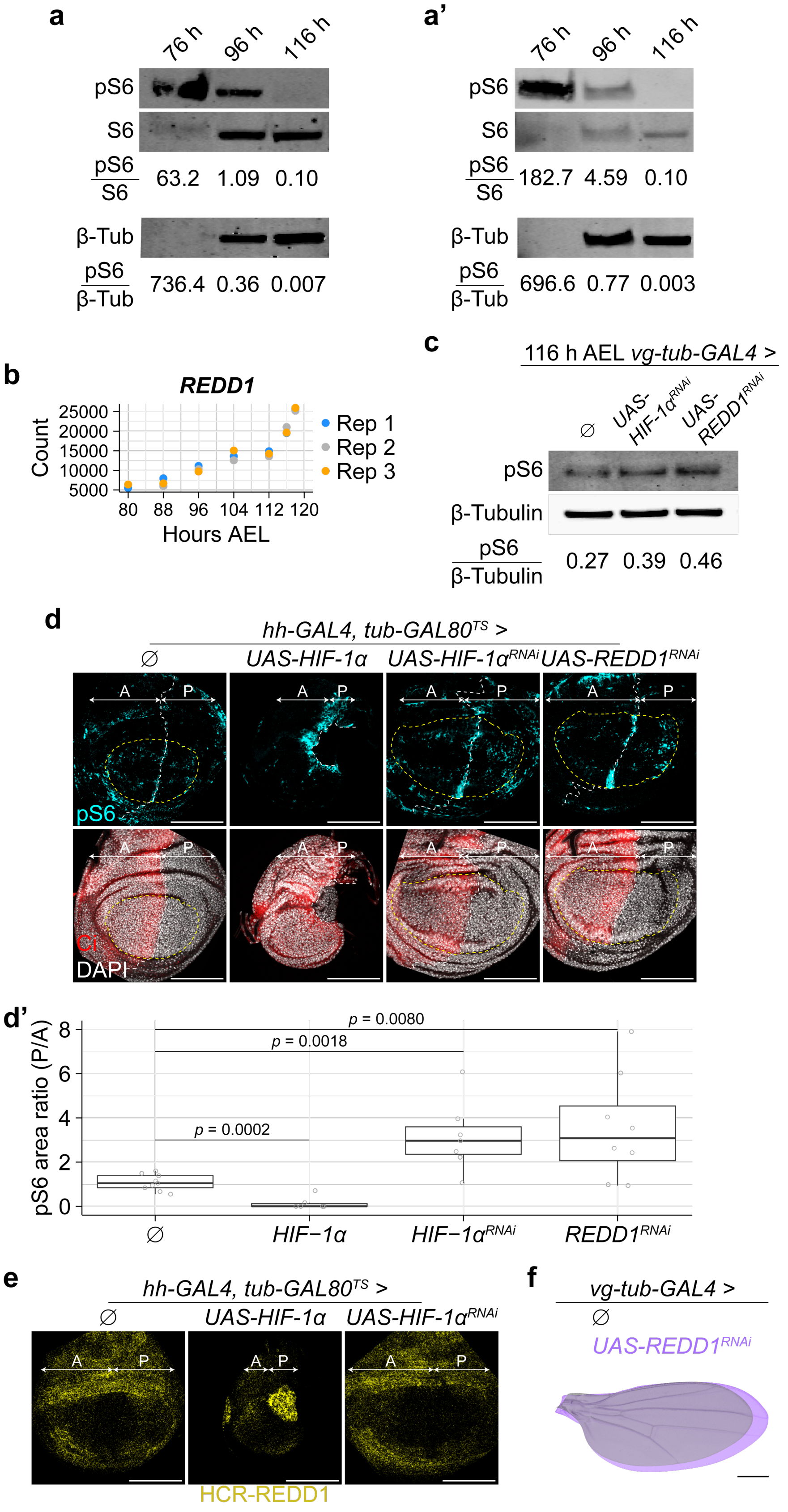

Supplementary Fig. 6: HIF-1α dampens growth by suppressing TOR activity through transcriptional activation of *REDD1*.

a-a'. Level of pS6 in whole wing discs decreases during L3. pS6 levels are normalised to total S6 or β-Tub with the ratio indicated under each lane. Two independent experiments are shown. Source data are provided as uncropped scans of blots and Excel sheets.
b. *REDD1* transcripts, detected by bulk RNA-Seq of L3 discs, decrease over time.
c-c'. pS6 does not decrease at the end of L3 upon whole-disc knockdown of *HIF-1α* or *REDD1.* pS6 levels are normalised to β-Tub, with the ratio indicated under each lane. Source data are provided as uncropped scans of blots and an Excel sheet.
d-d'. Changes in pS6 immunoreactivity in response to modulation of HIF-1α or REDD1 activity. Overexpression of HIF-1α in the P compartment (with *hh-GAL4*) dampens growth and reduces pS6, while knockdown of *HIF-1α* or *REDD1* enhances pS6 immunoreactivity. Representative images are shown in **d** and quantification of the immunofluorescence P/A ratio in the pouch is shown in **d'**. The A-P boundary was outlined manually with a dotted white line based on Ci staining. High pS6 immunoreactivity at the A-P boundary is also noticeable in wild-type discs, perhaps as a result of a discontinuity in Dpp signaling [2]. Scale bar = 100 μm. n $\geq$ 7 for each genotype, except for data of *HIF-1α* where n = 6. After confirming normality of the data, statistical significance was assessed by a two-sided unpaired t-test.
e. Changes in *REDD1* transcript level (detected by hybridization chain reaction) upon modulation of HIF-1α activity. HIF-1α overexpression in the P compartment boosts *REDD1* transcript, while *HIF-1α* knockdown causes a mild reduction. Scale bar = 100 μm.
f. *REDD1* knockdown leads to wing size increase as expected from an increase in TOR activity.

Supplementary Figure 7

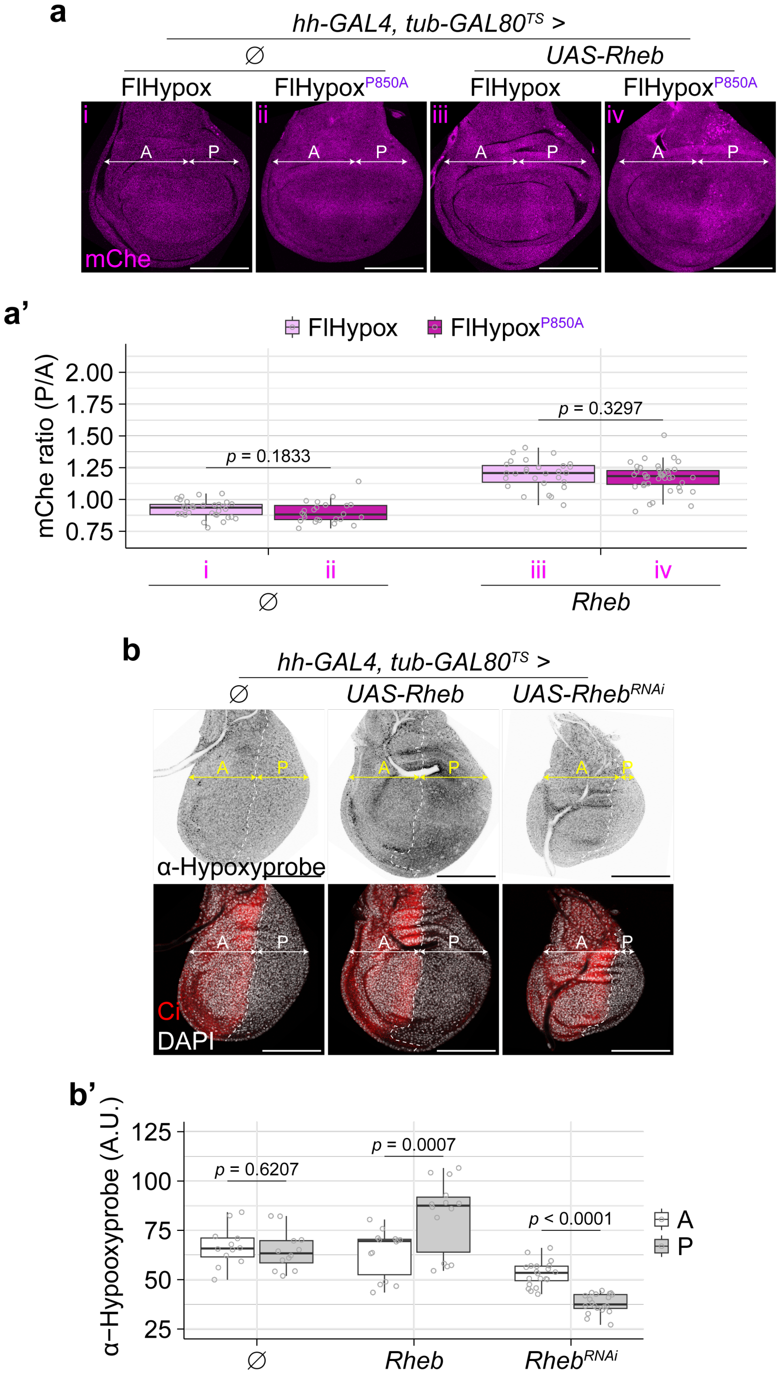

Supplementary Fig. 7: Effect of modulating TOR activity on *ubi-mCherry* and Hypoxyprobe^{TM} signal.

a-a'. Effect of Rheb overexpression on fluorescence from the *ubi-mCherry* module linked to FlHypox or FlHypox^{P850A}. Rheb overexpression in the P compartment induces a slight increase in the P/A ratio, showing that TOR signalling boosts the activity of this generic transgene, albeit not as much as that of FlHypox. Representative images are shown in **a** (all discs imaged under identical conditions) and fluorescence in the P compartment was normalised to that in the A compartment by calculating the P/A ratio plotted in **a'**. The images shown are of the same samples as those shown in **Fig. 4b**

b-b'. Fluorescence from Hypoxyprobe^{TM} staining is altered by modulation of Rheb levels. Larvae were reared at 18 °C on fly food for five days AEL, then shifted to 29 °C for 48 h to induce expression of *UAS* transgenes. During this induction period, larvae were maintained on fly food containing 1 mg/mL Hypoxyprobe^{TM}. Overexpression of Rheb in the P compartment (with *hh-GAL4*) enhances the Hypoxyprobe^{TM} signal. Knockdown of *Rheb* has the opposite effect. Representative images are shown in **b** (all discs imaged under identical conditions) and quantification of wing pouch fluorescence intensity is shown in **b'**. Scale bar = 100 μm. n $\geq$ 7 discs for each genotype, except for data for Ø (no *UAS* transgene), n = 6. Depending on normality of the data, statistical significance was assessed with either a two-sided unpaired t-test or a two-sided unpaired Wilcoxon signed-rank test.

Supplementary Figure 8

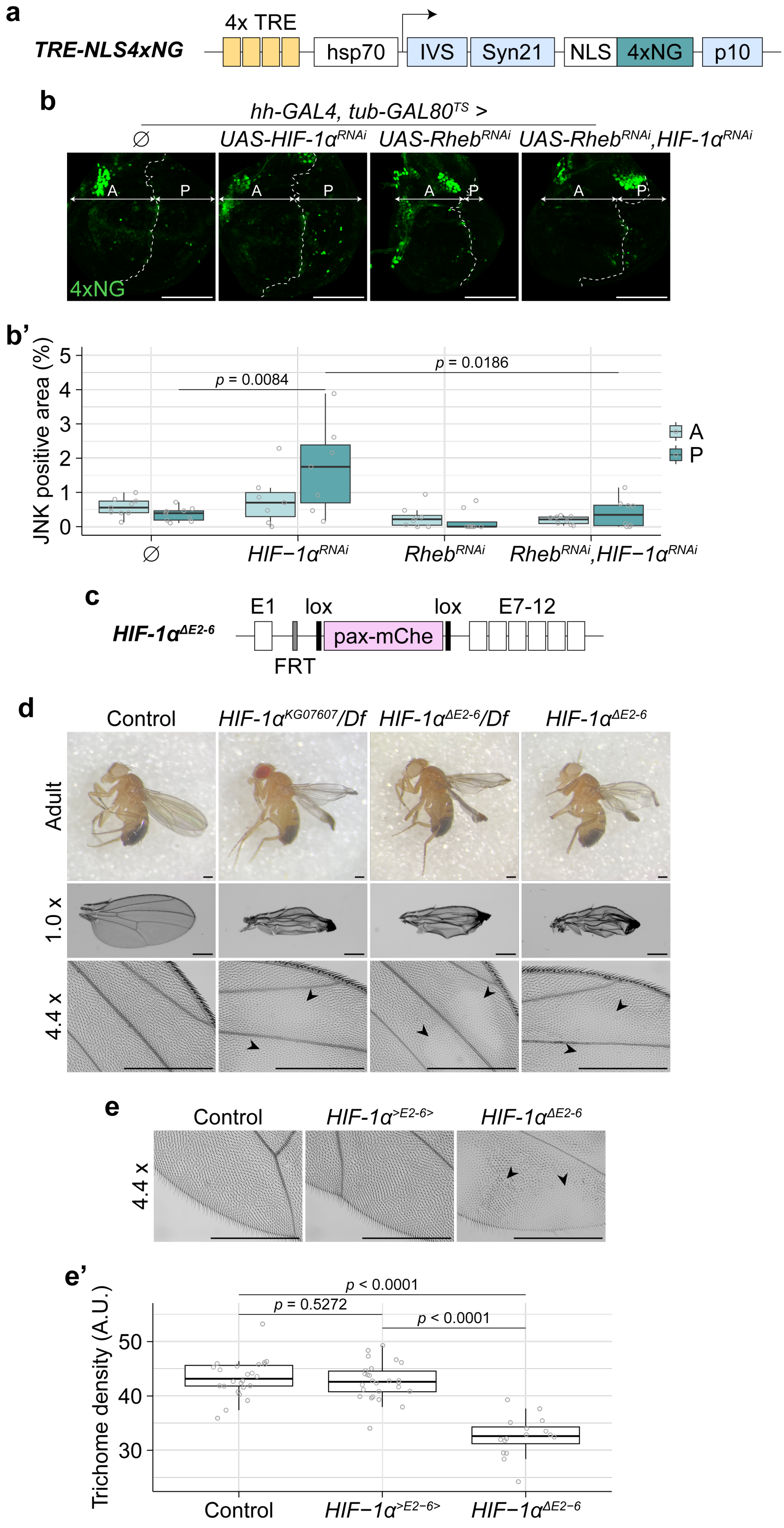

Supplementary Fig. 8: HIF-1α loss of function causes cellular stress and morphological defects.

a. Schematic representation of the *TRE-NLS4xNG* transgene to report JNK signalling. *TRE-NLS4xNG* was adapted from 12-O-Tetradecanoylphorbol-13-acetate (TPA) response element (TRE) reporters designed by Chatterjee and Bohmann [3]. TRE represents the consensus DNA binding of activator protein 1 (AP-1).
b-b'. Cellular stress induced by *HIF-1α* knockdown is reduced by reducing TOR activity. Knockdown of *HIF-1α* boosts JNK signalling (as shown in **Fig. 5a**), which is brought back down by knocking-down *Rheb* (right-hand panel). Representative images are shown in **b** (all discs imaged under identical conditions) and quantification of wing pouch fluorescence area ratio is shown in **b'**. Scale bar = 100 μm. n $\geq$ 8 discs for each genotype, except for data with *HIF-1*α^{RNAi} where n = 7. Depending on normality of the data, statistical significance was assessed by either a two-sided unpaired t-test or a two-sided unpaired Wilcoxon signed-rank test.
c. Schematic representation of the *HIF-1α* KO allele, *HIF-1*$\alpha^{\Delta E2\text{-}6}$. Exon 2 to exon 6 are replaced by a *pax-mCherry* module that can be used to track the allele.
d. Wing phenotypes of various *HIF-1α* mutant combinations. The observed phenotypes can be classified as either mild or severe. Mild phenotypes include flat wings with patchy trichome loss (black arrowheads), as well as altered trichome morphology and density (see **e-e'** below). Severe phenotypes include crumpled wings in addition to the trichome defects. Representative images are shown. Scale bar represents the same length in all panels. In *HIF-1*$\alpha^{\Delta E2\text{-}6}$ homozygotes, penetrance was 41.4% for mild and 58.6% for severe phenotypes (see **Fig. 5b**). Neither phenotype was observed in control wings.
e-e'. Trichome phenotypes of *HIF-1α* mutant wings. Representative images are shown in **e** and quantification of trichome density in **e'**. The severe phenotype is not included, as crumpled wings are difficult to image at high magnification. Black arrowheads point towards the absence of trichome or the abnormally long and soft trichomes. Scale bar represents the same length in all panels. n $\geq$ 24 wings for each genotype, except for data with *HIF-1*$\alpha^{\Delta E2\text{-}6}$ homozygotes where n = 16. After confirming normality of the data, statistical significance was assessed by a two-sided unpaired t-test.

Supplementary Figure 9

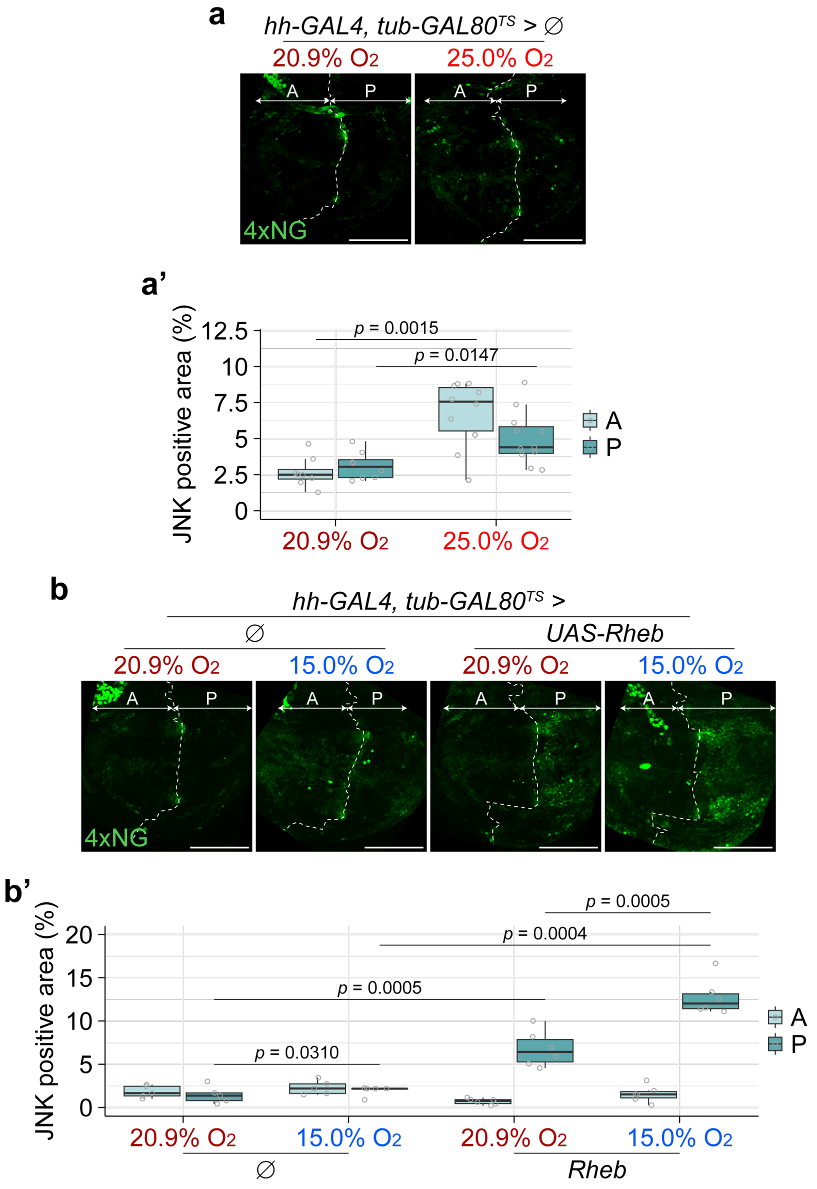

Supplementary Fig. 9: Effect of oxygen level on Rheb-induced cellular stress.

a-a’. Moderate hypoxia (25% oxygen) triggers JNK signalling (cellular stress). Larvae were cultured at the indicated oxygen tension for 48 h (see Methods) before discs were dissected out and processed for imaging. No ectopic expression was induced since no *UAS* transgene was present (∅). Representative images are shown in **a** (all discs imaged under identical conditions) and quantification of wing pouch fluorescence area ratio in **a’**. Scale bar = 100 μm. n $\geq$ 8 for each condition. Depending on normality of the data, statistical significance was assessed by either a two-sided unpaired t-test or a two-sided unpaired Wilcoxon signed-rank test.

b-b’. Cellular stress induced by excess TOR activity increases under mild hypoxia. Overexpression of Rheb in the P compartment (with *hh-GAL4*) increases cellular stress, and this is exacerbated by mild hypoxia at 15.0% oxygen (right-hand panel). Representative images are shown in **b** (all discs imaged under identical conditions) and quantification of wing pouch fluorescence per unit area is shown in **b’**. Scale bar = 100

μm. n $\geq$ 5 for each condition. Depending on normality of the data, statistical significance was assessed by either a two-sided unpaired t-test or a two-sided unpaired Wilcoxon signed-rank test.

References

1. White, K. P., Rifkin, S. A., Hurban, P. & Hogness, D. S. Microarray analysis of Drosophila development during metamorphosis. *Science* **286,** 2179–2184 (1999).
2. Romero-Pozuelo, J., Demetriades, C., Schroeder, P. & Teleman, A. A. CycD/Cdk4 and discontinuities in Dpp signaling activate TORC1 in the Drosophila wing disc. *Developmental cell* **42,** 376–387 (2017).
3. Chatterjee, N. & Bohmann, D. A versatile ΦC31 based reporter system for measuring AP-1 and Nrf2 signaling in Drosophila and in tissue culture. *PloS one* **7,** e34063 (2012).

Source Data for Supplementary Figure 6a

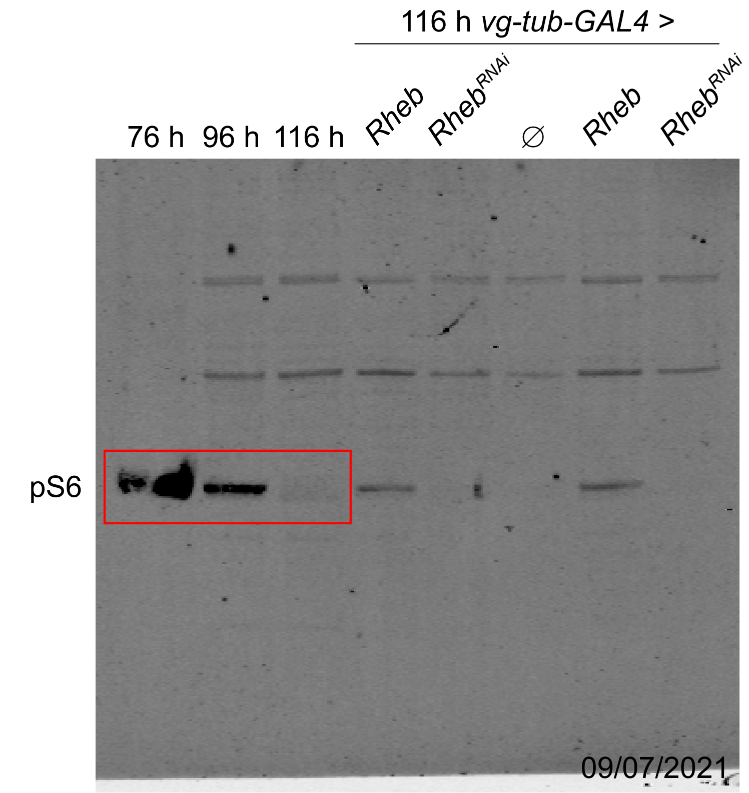

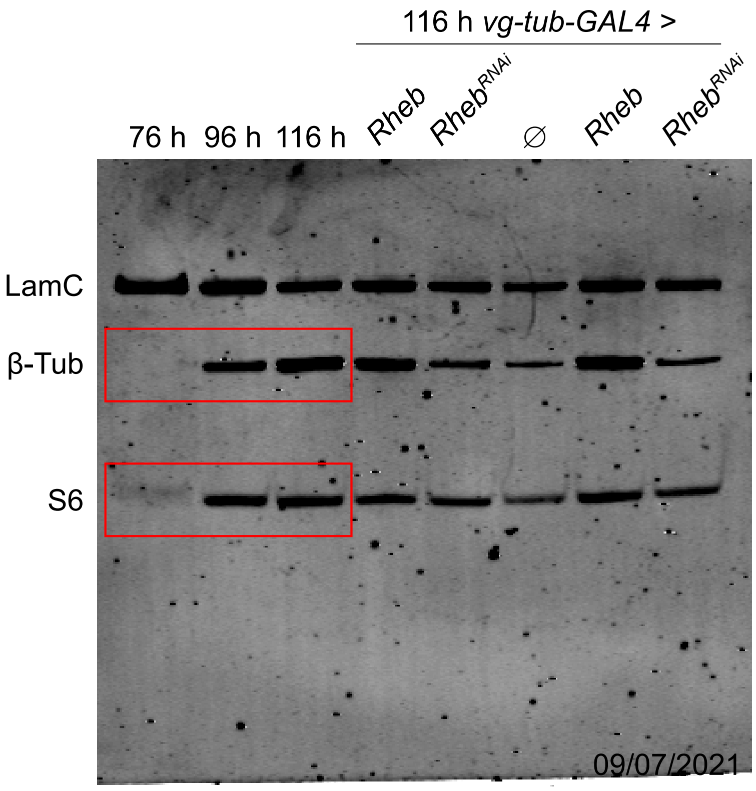

Source Data for Supplementary Figure 6a’

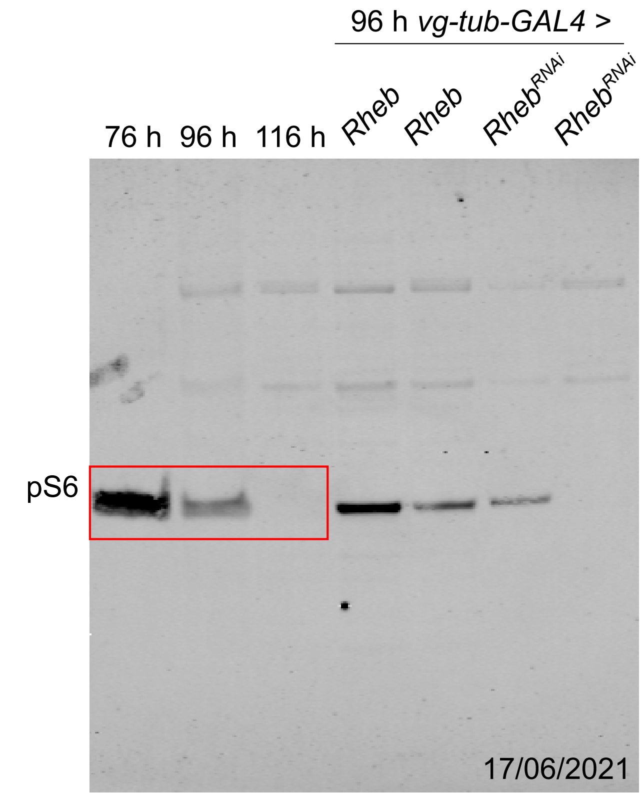

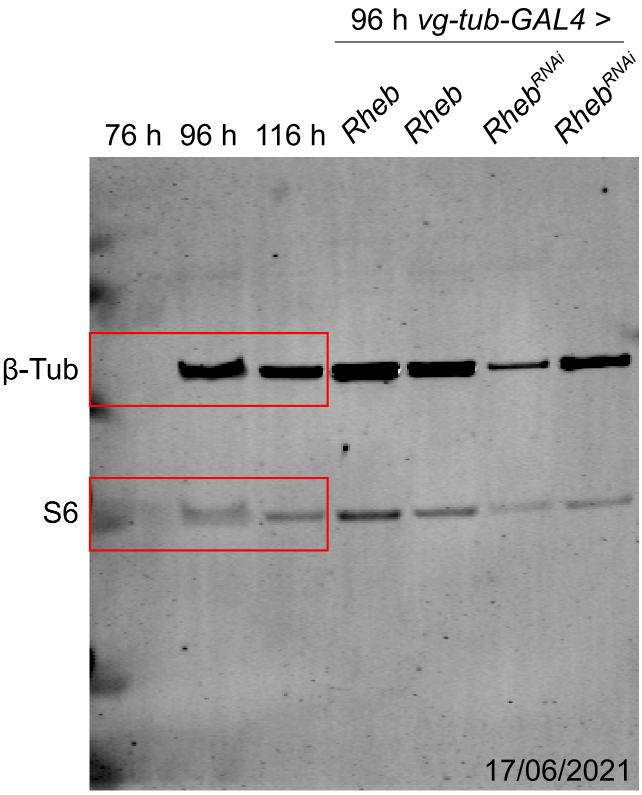

Source Data for Supplementary Figure 6c

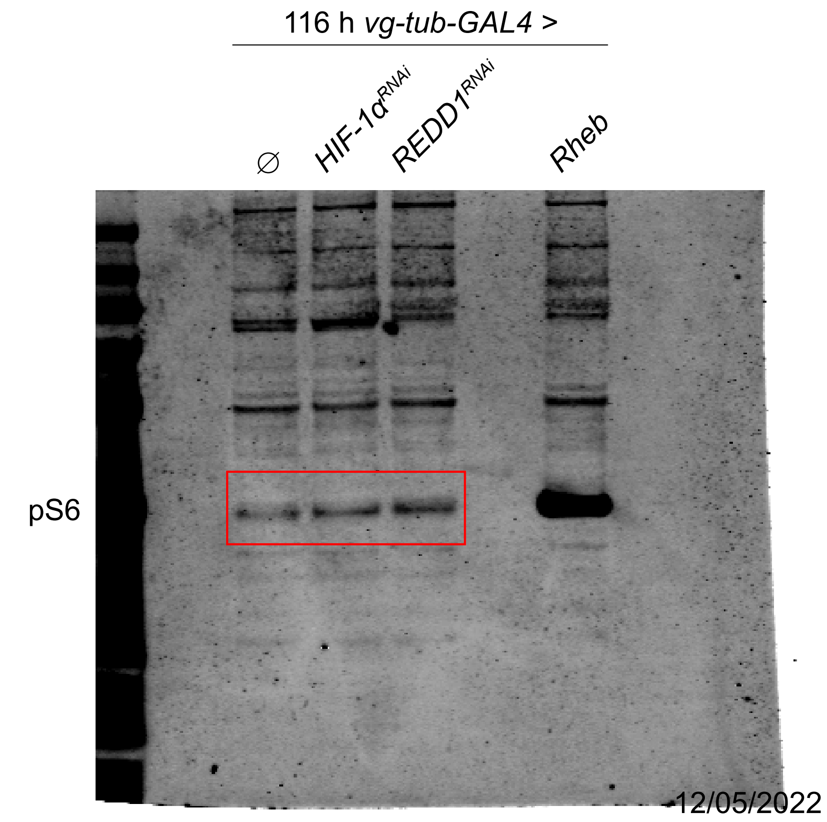

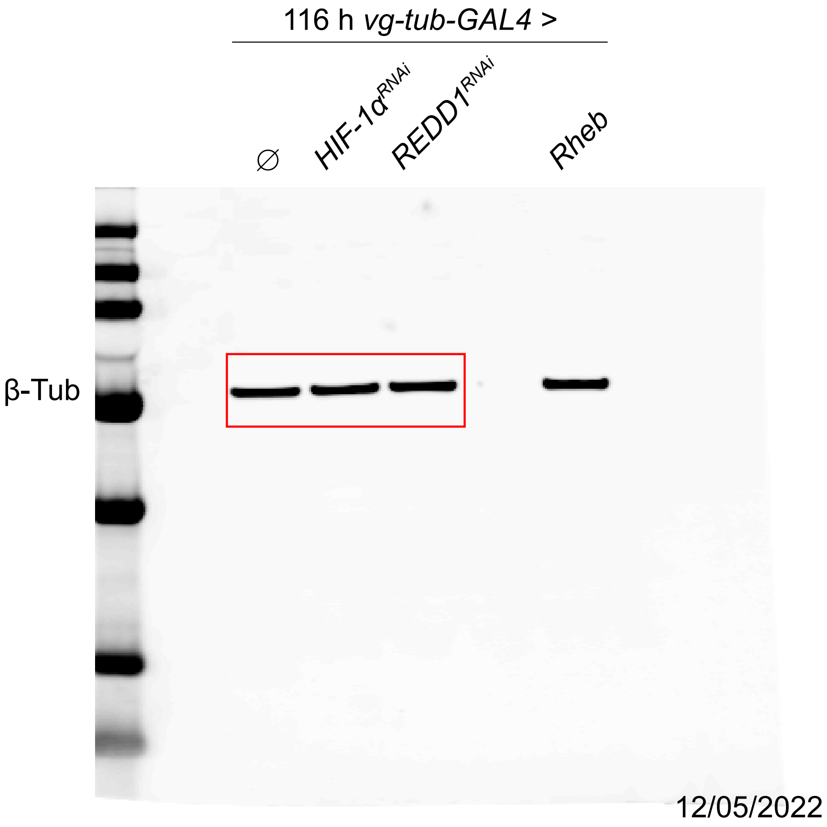

Supplement: Supplementary file 1 — Supplementary Information [file 41467_2025_67089_MOESM1_ESM.pdf]
