# Supplementary material for: HIF-1α-mediated feedback prevents TOR signalling from depleting oxygen supply and triggering stress during normal development
Source: Nat Commun. 2025 Dec 21;17:397. doi: 10.1038/s41467-025-67089-6 (PMC12796383; doi:10.1038/s41467-025-67089-6)
Supplement: Supplementary file 2 — Reporting summary [file 41467_2025_67089_MOESM2_ESM.pdf]

nature portfolio

Corresponding author(s): Jean-Paul Vincent

Last updated by author(s): Oct 2, 2025

Reporting Summary

Nature Portfolio wishes to improve the reproducibility of the work that we publish. This form provides structure for consistency and transparency in reporting. For further information on Nature Portfolio policies, see our Editorial Policies and the Editorial Policy Checklist.

Statistics

For all statistical analyses, confirm that the following items are present in the figure legend, table legend, main text, or Methods section.

n/a	Confirmed	
☐	☒	The exact sample size (*n*) for each experimental group/condition, given as a discrete number and unit of measurement
☐	☒	A statement on whether measurements were taken from distinct samples or whether the same sample was measured repeatedly
☐	☒	The statistical test(s) used AND whether they are one- or two-sided<br>*Only common tests should be described solely by name; describe more complex techniques in the Methods section.*
☒	☐	A description of all covariates tested
☐	☒	A description of any assumptions or corrections, such as tests of normality and adjustment for multiple comparisons
☐	☒	A full description of the statistical parameters including central tendency (e.g. means) or other basic estimates (e.g. regression coefficient) AND variation (e.g. standard deviation) or associated estimates of uncertainty (e.g. confidence intervals)
☐	☒	For null hypothesis testing, the test statistic (e.g. F, t, r) with confidence intervals, effect sizes, degrees of freedom and P value noted<br>*Give P values as exact values whenever suitable.*
☒	☐	For Bayesian analysis, information on the choice of priors and Markov chain Monte Carlo settings
☒	☐	For hierarchical and complex designs, identification of the appropriate level for tests and full reporting of outcomes
☐	☒	Estimates of effect sizes (e.g. Cohen's *d*, Pearson's *r*), indicating how they were calculated

Our web collection on statistics for biologists contains articles on many of the points above.

Software and code

Policy information about availability of computer code

Data collection	Leica LAS X was used for collecting confocal images. Fiji was used for imaging processing. RStudio was used for data analysis.
Data analysis	The raw reads of RNA-Sequencing were quantified using version 3.0 of NFCore's rnaseq pipeline (Nf-core/rnaseq: Nf-core/rnaseq v3.14.0 - hassium honey badger (3.14.0)), with star-rsem as the alignment method against release 86 of Ensembl's BDGP6 genome. Subsequently, these read counts were analysed using Bioconductor's package DESeq2 version 1.30.1 (Love et al, 2014, Moderated estimation of fold change and dispersion for RNA-seq data with DESeq2. Genome biology, 15, pp.1-21).

For manuscripts utilizing custom algorithms or software that are central to the research but not yet described in published literature, software must be made available to editors and reviewers. We strongly encourage code deposition in a community repository (e.g. GitHub). See the Nature Portfolio guidelines for submitting code & software for further information.

Data

Policy information about availability of data

All manuscripts must include a data availability statement. This statement should provide the following information, where applicable:

- Accession codes, unique identifiers, or web links for publicly available datasets
- A description of any restrictions on data availability
- For clinical datasets or third party data, please ensure that the statement adheres to our policy

The RNA-Seq data is publicly available at the Gene Expression Omnibus: GSE264119. FlyBase ID and names for the genes identified by RNA-Seq are available at

FlyBase (http://flybase.org/). All data supporting the findings of this study are available within the article or from the corresponding authors upon reasonable request.

Research involving human participants, their data, or biological material

Policy information about studies with human participants or human data. See also policy information about sex, gender (identity/presentation), and sexual orientation and race, ethnicity and racism.

Reporting on sex and gender	*Use the terms sex (biological attribute) and gender (shaped by social and cultural circumstances) carefully in order to avoid confusing both terms. Indicate if findings apply to only one sex or gender; describe whether sex and gender were considered in study design; whether sex and/or gender was determined based on self-reporting or assigned and methods used. Provide in the source data disaggregated sex and gender data, where this information has been collected, and if consent has been obtained for sharing of individual-level data; provide overall numbers in this Reporting Summary. Please state if this information has not been collected. Report sex- and gender-based analyses where performed, justify reasons for lack of sex- and gender-based analysis.*
Reporting on race, ethnicity, or other socially relevant groupings	*Please specify the socially constructed or socially relevant categorization variable(s) used in your manuscript and explain why they were used. Please note that such variables should not be used as proxies for other socially constructed/relevant variables (for example, race or ethnicity should not be used as a proxy for socioeconomic status). Provide clear definitions of the relevant terms used, how they were provided (by the participants/respondents, the researchers, or third parties), and the method(s) used to classify people into the different categories (e.g. self-report, census or administrative data, social media data, etc.) Please provide details about how you controlled for confounding variables in your analyses.*
Population characteristics	*Describe the covariate-relevant population characteristics of the human research participants (e.g. age, genotypic information, past and current diagnosis and treatment categories). If you filled out the behavioural & social sciences study design questions and have nothing to add here, write "See above."*
Recruitment	*Describe how participants were recruited. Outline any potential self-selection bias or other biases that may be present and how these are likely to impact results.*
Ethics oversight	*Identify the organization(s) that approved the study protocol.*

Note that full information on the approval of the study protocol must also be provided in the manuscript.

Field-specific reporting

Please select the one below that is the best fit for your research. If you are not sure, read the appropriate sections before making your selection.

☒ Life sciences ☐ Behavioural & social sciences ☐ Ecological, evolutionary & environmental sciences

For a reference copy of the document with all sections, see nature.com/documents/nr-reporting-summary-flat.pdf

Life sciences study design

All studies must disclose on these points even when the disclosure is negative.

Sample size	For RNA-Sequencing, three biological replicates were included for each time point, from 80 to 118 hours (h) after egg laying (AEL). For immunofluorescence imaging and Drosophila adult wing imaging, at least five samples were included in each experiment in order to perform statistical analysis, except Extended Data Fig. 5c where four samples were included. The exact sample volumes for each experiment are specified in the figure legends. Representative images are shown in figures. For Western blots, three biological replicates were included for each experiment.
Data exclusions	In Fig. 1e and 1f, one biological replicate at 80 h AEL were excluded from RNA-Sequencing as this RNA sample did not pass the quality control.
Replication	Experiments were performed in at least three independent biological replicates and all attempts at replication were successful, expect for the one RNA sample mentioned in Section Data exclusions.
Randomization	Randomization was not required for this study, as the phenotypes of samples with identical genotypes were reproducible.
Blinding	Blinding was not required for this study, as the phenotypes of experimental groups were distinctly different from the controls.

Reporting for specific materials, systems and methods

We require information from authors about some types of materials, experimental systems and methods used in many studies. Here, indicate whether each material, system or method listed is relevant to your study. If you are not sure if a list item applies to your research, read the appropriate section before selecting a response.

Materials & experimental systems

n/a	Involved in the study
☐	☒ Antibodies
☒	☐ Eukaryotic cell lines
☒	☐ Palaeontology and archaeology
☐	☒ Animals and other organisms
☒	☐ Clinical data
☒	☐ Dual use research of concern
☒	☐ Plants

Methods

n/a	Involved in the study
☒	☐ ChIP-seq
☒	☐ Flow cytometry
☒	☐ MRI-based neuroimaging

Antibodies

Antibodies used

Primary antibodies: mouse anti-V5 (1:500, Thermo Fisher Scientific R960-25), rabbit PAb2627 (Hypoxyprobe, Inc., HP PAb2627), rabbit anti-pS6 (1:5000 for Western blot, Kim et al, 2017), rabbit anti-pS6 (1:500 for immunofluorescence, Gui et al, 2023), rabbit anti-pS6 (1:100, this study), mouse anti-S6 (54D2) (1:100, CST #2317), rat anti-Ci (1:100, DSHB #2A1), mouse anti-β-Tubulin (1:500, DSHB #E7).
Alexa FluorTM Plus and Alexa FluorTM secondary antibodies raised in goat were obtained from Thermo Fisher Scientific and used at 1:1000 dilution (#A32728, #A32732, #A-21247). IRDye secondary antibodies raised in donkey were obtained from LI-COR Biotechnology and used at 1:10000 dilution (#926-32213, #926-68070).

Validation

Previous validations and citations can be found by using the RRID numbers. Rabbit anti-pS6 for Western blot was generated, validated and provided by Prof Jongkyeong Chung (Kim et al, 2017, Spatial Activation of TORC1 Is Regulated by Hedgehog and E2F1 Signaling in the Drosophila Eye. Developmental Cell, 42(4), pp.363-375). Rabbit anti-pS6 for immunofluorescence was generated, validated and provided by Dr Felipe Karam Teixeira (Gui et al, 2023, Simultaneous activation of Tor and suppression of ribosome biogenesis by TRIM-NHL proteins promotes terminal differentiation. Cell Reports, 42(3)). Rabbit anti-pS6 generated in this study was validated by ELISA and Western blot. CST #2317 (RRID: AB_2238583), DSHB #2A1 (RRID: AB_2109711), DSHB #E7 (RRID: AB_528499), #A32728 (RRID: AB_2633277), #A32732 (RRID: AB_2633281), #A-21247 (RRID: AB_141778), #926-32213 (RRID:AB_621848), #926-68070 (RRID:AB_10956588).

Animals and other research organisms

Policy information about studies involving animals; ARRIVE guidelines recommended for reporting animal research, and Sex and Gender in Research

Laboratory animals

Drosophila melanogaster L3 larvae and adults

Wild animals

N/A

Reporting on sex

Only male larvae were used in this study to elimiate potential sex-related effects.

Field-collected samples

N/A

Ethics oversight

Ethical approval was not required

Note that full information on the approval of the study protocol must also be provided in the manuscript.

Plants

Seed stocks

Report on the source of all seed stocks or other plant material used. If applicable, state the seed stock centre and catalogue number. If plant specimens were collected from the field, describe the collection location, date and sampling procedures.

Novel plant genotypes

Describe the methods by which all novel plant genotypes were produced. This includes those generated by transgenic approaches, gene editing, chemical/radiation-based mutagenesis and hybridization. For transgenic lines, describe the transformation method, the number of independent lines analyzed and the generation upon which experiments were performed. For gene-edited lines, describe the editor used, the endogenous sequence targeted for editing, the targeting guide RNA sequence (if applicable) and how the editor was applied.

Authentication

Describe any authentication procedures for each seed stock used or novel genotype generated. Describe any experiments used to assess the effect of a mutation and, where applicable, how potential secondary effects (e.g. second site T-DNA insertions, mosiacism, off-target gene editing) were examined.
